# Infection of the malaria mosquito, *Anopheles gambiae*, with two species of entomopathogenic fungi: effects of concentration, co-formulation, exposure time and persistence

**DOI:** 10.1186/1475-2875-8-309

**Published:** 2009-12-23

**Authors:** Ladslaus L Mnyone, Matthew J Kirby, Dickson W Lwetoijera, Monica W Mpingwa, Bart GJ Knols, Willem Takken, Tanya L Russell

**Affiliations:** 1 Biomedical and Environmental Group, Ifakara Health Institute, PO Box 53, Off Mlabani Passage, Ifakara, Tanzania; 2 Laboratory of Entomology, Wageningen University and Research Centre, PO Box 8031, 6700 EH, Wageningen, the Netherlands; 3 Department of Biological and Biomedical Sciences, University of Durham, South Road, Durham, DH1 3LE, UK; 4 Pest Management Centre, Sokoine University of Agriculture, PO Box 3110, Morogoro, Tanzania; 5 Department of Zoology and Marine Biology, University of Dar es Salaam, PO Box 35064, Dar es Salaam, Tanzania; 6 Division of Infectious Diseases, Tropical Medicine & AIDS Academic Medical Center, F4-217, Meibergdreef 9, 1105 AZ, Amsterdam, the Netherlands; 7 Vector Group, Liverpool School of Tropical Medicine, Liverpool, L3 5QA, UK

## Abstract

**Background:**

Entomopathogenic fungi *Metarhizium anisopliae *and *Beauveria bassiana *isolates have been shown to infect and reduce the survival of mosquito vectors.

**Methods:**

Here four different bioassays were conducted to study the effect of conidia concentration, co-formulation, exposure time and persistence of the isolates *M. anisopliae *ICIPE-30 and *B. bassiana *I93-925 on infection and survival rates of female *Anopheles gambiae sensu stricto*. Test concentrations and exposure times ranged between 1 × 10^7 ^- 4 × 10^10 ^conidia m^-2 ^and 15 min - 6 h. In co-formulations, 2 × 10^10 ^conidia m^-2 ^of both fungus isolates were mixed at ratios of 4:1, 2:1, 1:1,1:0, 0:1, 1:2 and 1:4. To determine persistence, mosquitoes were exposed to surfaces treated 1, 14 or 28 d previously, with conidia concentrations of 2 × 10^9^, 2 × 10^10 ^or 4 × 10^10^.

**Results:**

Mosquito survival varied with conidia concentration; 2 × 10^10 ^conidia m^-2 ^was the concentration above which no further reductions in survival were detectable for both isolates of fungus. The survival of mosquitoes exposed to single and co-formulated treatments was similar and no synergistic or additive effects were observed. Mosquitoes were infected within 30 min and longer exposure times did not result in a more rapid killing effect. Fifteen min exposure still achieved considerable mortality rates (100% mortality by 14 d) of mosquitoes, but at lower speed than with 30 min exposure (100% mortality by 9 d). Conidia remained infective up to 28 d post-application but higher concentrations did not increase persistence.

**Conclusion:**

Both fungus isolates are effective and persistent at low concentrations and short exposure times.

## Background

The control tools currently deployed to reduce malaria transmission in Africa are early diagnosis and prompt treatment, insecticide-treated bed nets (ITNs) and indoor residual spraying (IRS) [1,2]. Although these strategies have delivered reductions in childhood disease [3,4], there remains a high incidence of malaria in many countries, with over 300 - 500 million infections and 1 million deaths each year [5]. The factors responsible for continuing transmission include the development of *Plasmodium *resistance to drugs [6], *Anopheles *resistance to insecticides [7,8], and socio-economic or cultural resistance to control measures [9]. Clearly, as effective as the current tools are, they are not sufficient on their own to eliminate malaria from intensely endemic regions [10]. Therefore, additional control tools are needed to combat this disease.

Laboratory and small-scale household studies have demonstrated a great potential to develop entomopathogenic fungi for field control of malaria vectors [11,12]. The fungi penetrate the mosquito cuticle through mechanical pressure and/or enzymatic degradation of major cuticle components [13,14]. Once inside the host, the fungi propagate, consuming nutrients and releasing metabolites resulting in mycosis and death [15]. In the laboratory, the entomopathogenic fungus, *Beauveria bassiana *was able to reduce the number of adult anopheline mosquitoes capable of transmitting malaria by a factor of approximately 80 [16], and one field study on a household scale has shown that *Metarhizium anisopliae *ICIPE-30 can cause a two-fold reduction in the life span of adult mosquitoes [17].

Before entomopathogenic fungi can be scaled-up for use in biological control programmes, it is essential to determine the optimum concentration of conidia (asexual spores shed at maturity) to apply, whether co-formulations offer any advantage over single isolate applications, the exposure time required for conidia to infect mosquitoes, and whether there is a relationship between concentration and persistence. The optimum concentration will be identified as the lowest concentration that is able to achieve the maximum reduction in survival time for each fungal species. Co-formulations could have a synergistic or additive effect due to the different life histories of the fungi involved, or inter-species interactions such as competition-altering activity. Determining the minimum exposure time for infection will guide in developing realistic field dissemination tools having pre-defined whether the intervention can target either host-seeking or resting mosquitoes or both. Examining persistence will allow re-application rates to be defined. Holistically, this information will provide a sound indication on the viability of this technology for field control of mosquitoes. The study was, therefore, designed to address these issues in assays of the fungal isolates *M. anisopliae *ICIPE-30 and *B. bassiana *I93-925 against adult *Anopheles gambiae sensu stricto*. These isolates were chosen because of their proven efficacy, availability in the market, and minimum risks to non-targeted organisms.

## Methods

### Mosquito rearing and maintenance

*Anopheles gambiae *s.s. mosquitoes were reared at the Ifakara Health Institute (IHI), Tanzania. The colony was established from a population caught near Njage village, 70 km from Ifakara, in 1996. Larval and adult stages of the mosquitoes were raised using methods based on those described by the Huho *et al *[18]. Bioassays were conducted using 3-6 d old non blood fed adult female mosquitoes. During all experiments, mosquitoes were supplied 9% glucose solution.

### Fungal isolates, formulation and application

Two entomopathogenic fungi species were used in all bioassays: 1) *Metarhizium anisopliae var. anisopliae *isolate ICIPE-30, isolated originally in 1989 from the maize stalk borer, *Busseola fusca *(Lepidoptera, Noctuidae) in Western Kenya, and imported as dry conidia from Wageningen University, The Netherlands and 2) *Beauveria bassiana *isolate 193-825 (IMI 391510), isolated from a chrysomelid beetle (Coleoptera) in the USA and imported as dry conidia from the Commonwealth Scientific and Industrial Research Organization (CSIRO), Australia and Penn State University, USA. Before use each batch of conidia was checked for viability by inoculation on Sabouraud dextrose agar (SDA) plates, and only conidia with ≥ 85% germination were used in bioassays.

The conidia were formulated in oil for application. Oil protects conidia from adverse environmental conditions and facilitates adhesion to the insect cuticle. Initially, a fungal stock solution was prepared by suspending 1-2 g of conidia in 20 ml of highly refined mineral oil (Shell Ondina 917^®^, Shell, The Netherlands) or Enerpar (Enerpar M002^®^, BP Southern Africa Ltd). To homogenize the mixture it was shaken vigorously, vortexed for 25 sec and then sonicated (ultrasonic bath, Langford Electronics, UK) for 3 min. Dilutions of 1:10, 1:100 and 1:1000 were prepared in oil and the concentration of conidia was calculated using a Neubauer Haemocytometer (Hausser Scientific, USA) with the aid of a compound microscope (Leica ATC2000, USA) at 400× magnification. The solution was adjusted to the desired concentrations for application by diluting with mineral oil. Neither Ondina nor Enerpar oils had any negative effect on conidia germination or mosquito mortality (unpublished data). Unless otherwise stated, the conidia used during the bioassays were formulated in Ondina oil.

Mosquitoes were exposed to conidia applied to sheets of A4 printing paper within plastic exposure tubes (8.2 cm diameter × 12.5 cm height), closed with netting also treated with conidia. The paper and netting were treated using a hand-held pressure sprayer (Minijet^®^, SATA, Germany) operating at a constant pressure of 2 bars. The nozzle of the spray gun was held 50 cm away from, and at a right angle to, the application surface. A working solution of 23 ml containing the desired conidia concentration was applied to a 1 m^2 ^area. Treated surfaces were left to dry for 24 h. To avoid cross-contamination, formulations of each fungal isolate were applied in separate rooms.

### Bioassay procedures

30-50 adult *An. gambiae s.s*. mosquitoes were introduced to the exposure tubes and held for 6 h (unless stated otherwise in bioassay descriptions below), after which they were transferred to separate untreated cages (9 cm^3^) and maintained at 26-27°C and 85-95% relative humidity (RH) with access to 9% glucose solution *ad libitum*. Survival was monitored daily for a maximum of 28 d. Dead mosquitoes were collected individually and put onto moist filter paper in Petri dishes, sealed with parafilm, and incubated at 26-27°C and 85-95% RH for 3-4 d, after which they were examined for evidence of fungal sporulation basing on colour of their conidia. *Metarhizium anisopliae *yields green conidia, whereas, *B. bassiana *yields white conidia. Similar bioassay procedure was carried out for 30-50 mosquitoes in control groups, except that they were exposed to untreated surfaces. During all of the bioassays, four replicates were used for each experimental factor. Four different bioassays were conducted to study the effect of 1) concentration, 2) co-formulation 3) exposure time and 4) persistence on infection and survival of *A. gambiae s.s*.

### Bioassay 1: concentration

Mosquitoes were exposed to six different concentrations of the two fungal isolates: (1 × 10^7^, 2 × 10^8^, 1 × 10^9^, 2 × 10^9^, 2 × 10^10 ^and 4 × 10^10 ^conidia m^-2^). Concentrations were chosen basing on their reported efficacy against mosquitoes and other arthropods, and likelihood of being cost effective.

### Bioassay 2: co-infection with *M. anisopliae *and *B. bassiana*

Mosquitoes were exposed to co-formulations of 2 × 10^10 ^conidia m^-2^of both fungus isolates mixed at ratios of 4:1, 2:1, 1:1, 1:0, 0:1, 1:2 and 1:4.

### Bioassay 3: exposure time

Mosquitoes were exposed to 2 × 10^10 ^conidia m^-2 ^for four different lengths of time: 15 min, 30 min, 1 and 6 h. In a separate experiment two concentrations, 2 × 10^10 ^and 4 × 10^10 ^conidia m^-2^, formulated in Enerpar oil, were evaluated at 15 and 30 min exposure to determine whether concentration affected time required for infection.

### Bioassay 4: persistence

The residual activity of conidia was determined by exposing mosquitoes to the same treated surfaces 1, 14 and 28 d post application. Treated surfaces were kept at 26-27°C and 85-95% RH in-between exposure rounds. Three different concentrations formulated in Enerpar oil (2 × 10^9^, 2 × 10^10 ^and 4 × 10^10 ^conidia m^-2^).

### Statistical analysis

Mosquito survival data were analysed by Kaplan-Meier pair wise comparison using SPSS version 15. Data were stratified by replicate and multiple chi-square pair-wise comparisons were used to examine the effect of treatment on mosquito survival. Survival curves were considered not statistically different at *p *> 0.05. A probit regression (R-package version 2.9.1) was used to determine the concentration of conidia required for 50% and 90% mortality 10 d after exposure (lethal concentration: LC_50 _and LC_90_, Bioassay 1).

## Results

### Bioassay 1: concentration

**Figure 1:**
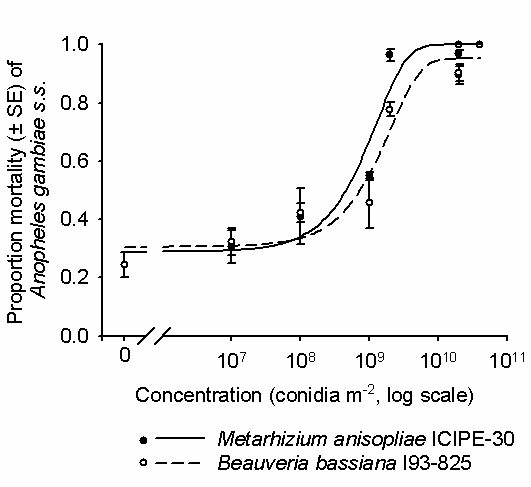
**The percentage mortality of *An. gambiae *s.s. mosquitoes 3 - 6 d of age, 10 days post exposure to different concentrations of *M. anisopliae *ICIPE-30 and *B. bassiana *I93-825**. Controls were not exposed to any fungus ('0' concentration). Mosquitoes were exposed to the treatments for 6 h. The sigmoidal models were fitted to the data using probit regression.

**Figure 2:**
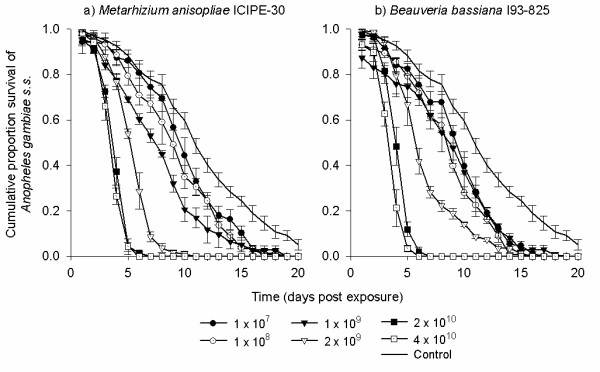
**The survival of *Anopheles gambiae *s.s. females after 6 h exposure to different concentrations (1 × 10^7^, 1 × 10^8^, 1 × 10^9^, 2 × 10^9^, 2 × 10^10 ^and 4 × 10^10 ^conidia m^-2^) of a) *Metarhizium anisopliae *ICIPE-30 and b) *Beauveria bassiana *I93-825**.

Concentrations of 2 × 10^9 ^conidia m^-2 ^and above of both *M. anisopliae *ICIPE-30 and *B. bassiana *I93-825 resulted in 85-95% mortality of exposed mosquitoes after 10 d. This was higher than 36 ± 3.4% mortality recorded from untreated control after 10 d (Figure 1). The daily survival of mosquitoes exposed to any fungal concentration was significantly reduced compared to that of controls (*P *< 0.001; Figure 1; Table 1). The median survival time (MST ± SE) of control mosquitoes was 11 ± 0.06 d. The MST of fungus-exposed mosquitoes was similar for both isolates and ranged between 4.0 ± 0.09 d at 4 × 10^10^ conidia m^-2 ^to 10 ± 0.32 d at 1 × 10^7 ^for *M. anisopliae*, and 4.0 ± 0.08 d at 4 × 10^10 ^to 10 ± 0.467 d at 1 × 10^7 ^for *B. bassiana*. There was no difference between 2 × 10^10 ^and 4 × 10^10 ^for *M. anisopliae *(4.0 ± 0.11, 4.0 ± 0.09 d, *X*^2 ^= 3.54, *P *= 0.07) or *B. bassiana *(4.0 ± 0.12, 4.0 ± 0.08 d, *X*^2 ^= 3.56, *P *= 0.06; Table 1). As such, optimum reduction in survival was considered to be reached at a concentration of 2 × 10^10 ^conidia m^-2 ^(Figure 2). The concentrations of conidia that were modelled to result in 50% and 90% (LC_90_) mortality were 1.02 × 10^7 ^(LC_50_) and 9.77 × 10^8 ^conidia m^-2 ^(LC_90_) *M. anisopliae *and 7.71 × 10^7 ^(LC_50_) and 2.66 × 10^9 ^conidia m^-2 ^(LC_90_) for *B. bassiana*.

**Table 1:** Kaplan Meier pair-wise comparisons of the median survival times (MST) of *An. gambiae *s.s. females exposed to different concentrations (1 × 10^7^, 1 × 10^8^, 1 × 10^9^, 2 × 10^9^, 2 × 10^10 ^and 4 × 10^10 ^conidia m^-2^) of *M. anisopliae *ICIPE-30 and *B. bassiana *I93-825 (IMI 391510) for 6 h.

Concentrations	MST ± SE	1 × 10^7^	1 × 10^8^	1 × 10^9^	2 × 10^9^	2 × 10^10^	4 × 10^10^
***M. anisopliae***							
**Control**	11 ± 0.32	χ^2 ^= 15.12	χ^2 ^= 24.00	χ^2 ^= 50.7	χ^2 ^= 195.11	χ^2 ^= 239.68	χ^2 ^= 261.26
		*p *< 0.001*	*p *< 0.001*	*p *< 0.001*	*p *< 0.001*	*p *< 0.001*	*P *< 0.001*
**1 × 10^7^**	10 ± 0.32		χ^2 ^= 1.87	χ^2 ^= 14.46	χ^2 ^= 138.37	χ^2 ^= 197.25	χ^2 ^= 219.10
			*p *= 0.17	*p *< 0.001*	*p *< 0.001*	*p *< 0.001*	*p <*0.001*
**1 × 10^8^**	9 ± 0.37			χ^2 ^= 5.95	χ^2 ^= 98.86	χ^2 ^= 166.80	χ^2 ^= 188.48
				*p <*0.001*	*p <*0.001*	*p *< 0.001*	*p *< 0.001*
**1 × 10^9^**	8 ± 0.43				χ^2 ^= 53.99	χ^2 ^= 119.57	χ^2 ^= 104.37
					*p *< 0.001*	*p <*0.001*	*p *< 0.001*
**2 × 10^9^**	6 ± 0.15					χ^2 ^= 79.62	χ^2 ^= 104.37
						*p *< 0.001*	*p *< 0.001*
**2 × 10^10^**	4 ± 0.12						χ^2 ^= 3.54
							*p = *0.07

***B. bassiana***							
**Control**	11 ± 0.32	χ^2 ^= 25.09	χ^2 ^= 47.61	χ^2 ^= 27.13	χ^2 ^= 109.37	χ^2 ^= 251.31	χ^2 ^= 251.60
		*p *< 0.001*	*p *< 0.001*	*p *< 0.001*	*p *< 0.001*	*p <*0.001*	*p *< 0.001*
**1 × 10^7^**	10 ± 0.47		χ^2 ^= 4.53	χ^2 ^= 0.319	χ^2 ^= 38.97	χ^2 ^= 171.18 *p*	χ^2 ^= 181.16
			*p *< 0.001*	*p = *0.57	*p *< 0.001*	*<*0.001*	*p *< 0.001*
**1 × 10^8^**	9 ± 0.23			χ^2 ^= 1.47	χ = 21.40	χ^2 ^= 158.71 *p*	χ^2 ^= 168.32
				*p *= 0.22	*p *< 0.001*	*<*0.001*	*p <*0.001*
**1 × 10^9^**	9 ± 0.58				χ^2 ^= 21.90	χ^2 ^= 112.07 *p*	χ^2 ^= 114.26
					*p *< 0.001*	<0.001*	*p *< 0.001*
**2 × 10^9^**	6 ± 0.17					χ^2 ^= 88.29	χ^2 ^= 130.05
						*p *< 0.001*	*p *< 0.001*
**2 × 10^10^**	4 ± 0.11						χ^2 ^= 3.56
							*p *= 0.06

*Significant at *p *< 0.05

### Bioassay 2: co-infection with *M. anisopliae *and *B. bassiana*

**Figure 3:**
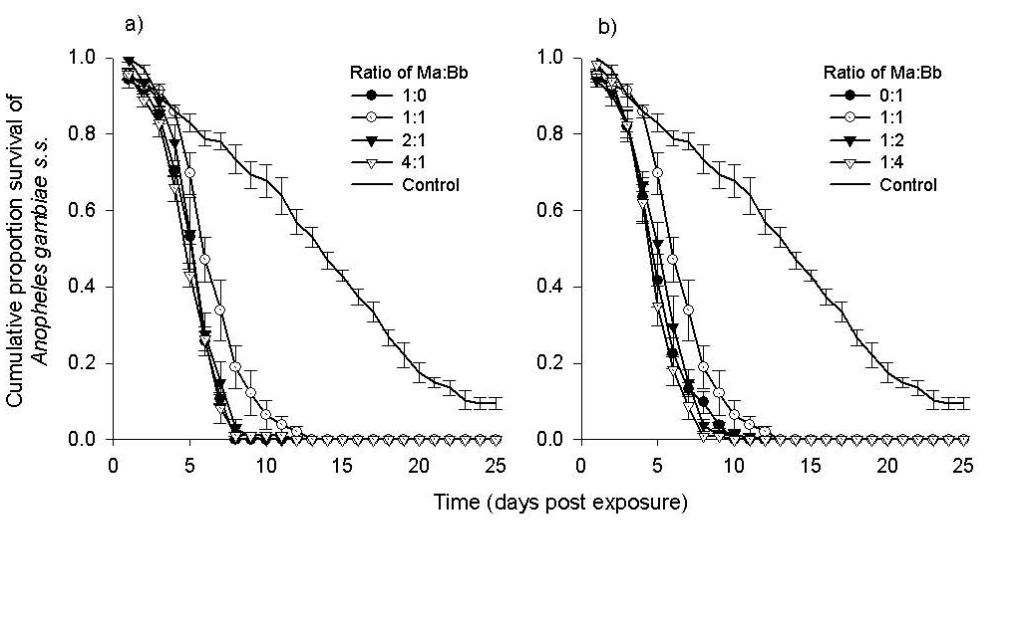
**The survival of *Anopheles gambiae *s.s. females after exposure to 2 × 10^10 ^conidia m^-2 ^of co-formulated *Metarhizium anisopliae *ICIPE-30 and *Beauveria bassiana *I93-825 under laboratory conditions**. The co-formulation was applied at different ratios of conidia (1:0, 1:1, 2:1 and 4:1) of *M. anisopliae *to *B. bassiana*.

The survival of mosquitoes exposed to co-formulated conidia was significantly reduced compared with control mosquitoes (*P *< 0.001, Figure 3, Table 2). However, survival was not further reduced compared with the single fungus strain (*P >*0.001, Table 2). The MST of mosquitoes exposed to co-formulations and single species formulations ranged between 5 - 6 d, while that of untreated controls was 13 d.

**Table 2:** Kaplan-Meier pair wise comparisons of the median survival times (MST) of *An. gambiae *s.s. females exposed to 2 × 10^10 ^conidia m^-2 ^of co-formulated *Metarhizium anisopliae *ICIPE-30 and *Beauveria bassiana *I93-825.

Ratio of Ma:Bb	MST ± SE	1:0	1:1	2:1	4:1
**Increasing ratio of *M. anisopliae***					
**Control**	13 ± 0.73	χ^2 ^= 132.57	χ^2 ^= 109.63	χ^2 ^= 134.20	χ^2 ^= 121.23
		*p *< 0.001*	*p *< 0.001*	*p *< 0.001*	*p *< 0.001*
**1:0**	6 ± 0.12		χ^2 ^= 0.15	χ^2 ^= 0.29	χ^2 ^= 1.37
			*p *= 0.70	*p *= 0.60	*p *= 0.24
**1:1**	6 ± 0.21			χ^2 ^= 0.65	χ^2 ^= 2.59
				*p *= 0.34	*p *= 0.07
**2:1**	6 ± 0.16				χ^2 ^= 0.53
					*p *= 0.46

**Increasing ratio of *B. bassiana***					
**Control**	13 ± 0.73	χ^2 ^= 128.01	χ^2 ^= 109.63	χ^2 ^= 133.22	χ^2 ^= 114.34
		*p *< 0.001*	*p *< 0.001*	*p *< 0.001*	*p *< 0.001*
**0:1**	5 ± 0.19		χ^2 ^= 1.63	χ^2 ^= 0.004	χ^2 ^= 0.29
			*p *= 0.20	*p *= 0.93	*p *= 0.59
**1:1**	6 ± 0.21			χ^2 ^= 0.29	χ^2 ^= 3.10
				*p *= 0.59	*p *= 0.08
**1:2**	6 ± 0.19				χ^2 ^= 3.24
					*p *= 0.06

Note: The co-formulation was applied at different ratios of conidia (1:0, 1:1, 2:1 and 4:1) of *M. anisopliae *to *B. bassiana*.

In all the co-formulation treatments, sporulation of both fungus species from the same cadaver was never observed. *Metarhizium anisopliae *sporulation only was observed in 78.17% (376/481) of the mosquito cadavers. No sporulation was observed on control mosquitoes.

### Bioassay 3: exposure time

**Figure 4:**
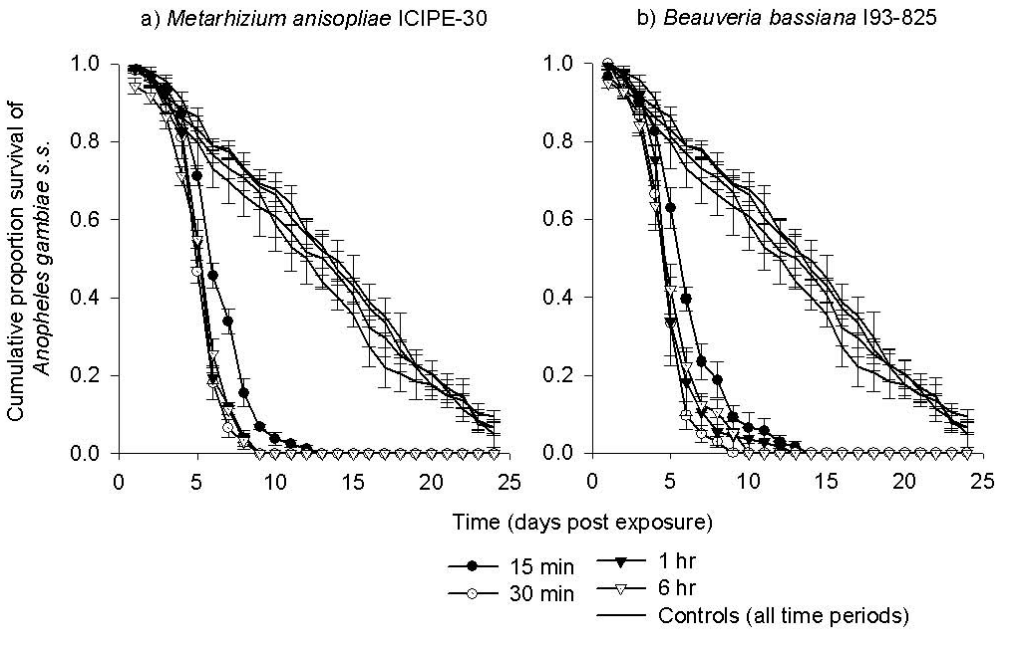
**The survival of *Anopheles gambiae *s.s. females after exposure to 2 × 10^10 ^conidia m^-2 ^of a) *Metarhizium anisopliae *ICIPE-30 and b) *Beauveria bassiana *I93-825 for different times (15 min, 30 min, 1 h and 6 h)**.

The exposure concentration used (2 × 10^10^) was selected on the basis of Bioassay 1 results. The ability of conidia of both fungi to kill and reduce the survival of mosquitoes was dependent on the length of exposure. The MST of mosquitoes after a 30 min exposure and above was significantly lower than that after 15 min for both *M. anisopliae *(*P *< 0.001) and *B. bassiana *(*P *< 0.001, Figure 4, Table 3). However, considerable mosquito mortality was still achieved with 15 min exposure (100% by 14 d), but at lower speed than with 30 min exposure (100% by 9 d). Nonetheless, 93.52% and 96.22% mortality was recorded when mosquitoes were exposed for 15 min to *B. bassiana *and *M. anisopliae *by 9 d. The MST for fungus-exposed mosquitoes ranged between 5 - 6 d. Survival of mosquitoes exposed to either *M. anisopliae *or *B. bassiana *for 15 min and above was significantly reduced as compared to controls (*P *< 0.001, Table 3).

**Table 3:** Kaplan-Meier pair wise comparisons of the median survival times (MST) of *An. gambiae *s.s. females exposed to *M. anisopliae *ICIPE-30 and *B. bassiana *I93-825 (IMI 391510) for different exposure times (15 min, 30 min, 1 h and 6 h).

Exposure time	MST ± SE	15 min	30 min	1 h	6 h
***M. anisopliae***					
**Control**	13 ± 0.32	χ^2 ^= 124.1	χ^2 ^= 154.65	χ^2 ^= 149.59	χ^2 ^= 150.98
		*p *< 0.001*	*p *< 0.001*	*p <*0.001*	*p *< 0.001*
**15 min**	6 ± 0.32		χ^2 ^= 36.89	χ^2 ^= 26.07	χ = 28.39
			*p *< 0.001*	*p <*0.001*	*p *< 0.001*
**30 min**	5 ± 0.37			χ^2 ^= 1.15	χ^2 ^= 0.57
				*p *= 0.28	*p *= 0.44
**1 h**	5 ± 0.43				χ^2 ^= 0.86
					*p *= 0.77

***B. bassiana***					
**Control**	13 ± 0.32	χ^2 ^= 118.5	χ^2 ^= 97.98	χ^2 ^= 79.84	χ^2 ^= 103.81
		*p *< 0.001*	*p *< 0.001*	*p *< 0.001*	*p *< 0.001*
**15 min**	6 ± 0.17		χ^2 ^= 42.68	χ^2 ^= 16.28	χ^2 ^= 14.97
			*p *< 0.001*	*p *< 0.001*	*p *< 0.001*
**30 min**	5 ± 0.11			χ^2 ^= 0.50,	χ^2 ^= 0.29
				*p *= 0.23	*p *= 0.59
**1 h**	5 ± 0.10				χ^2 ^= 0.06
					*p *= 0.81

*Significant at *p *< 0.05

In separate bioassay where conidia concentrations of 2 × 10^10 ^and 4 × 10^10 ^were compared, at each exposure time; 15 and 30 min, the two concentrations equally reduced mosquito survival. This was observed for *M. anisopliae *(*X*^2 ^= 0.63 - 2.92, *P *> 0.05) and *B. bassiana *(*X*^2 ^= 0.76 - 5.23, *P *> 0.05), [see Additional file 1].

### Bioassay 4: persistence

**Figure 5:**
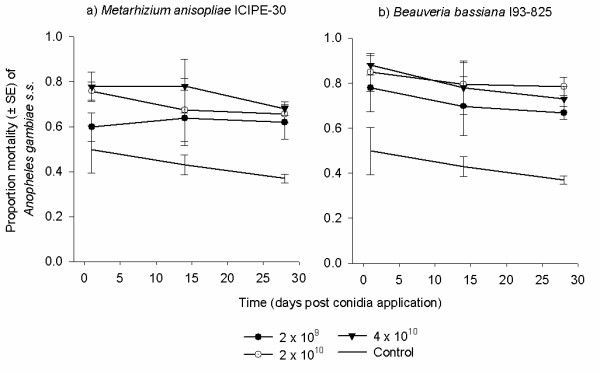
**Percentage mortality of mosquitoes 12 d post exposure to different concentrations (2 × 10^9^, 2 × 10^10 ^and 4 × 10^10 ^conidia m^-2^) of a) *Metarhizium anisopliae *ICIPE-30 and b) *Beauveria bassiana *I93-825 after storage of the treated materials for 1, 14 and 28 d**. Treated materials were stored under 26°C-27°C temperature and 85-95% humidity before successive re-exposures.

Overall, effect of *M. anisopliae *(*P *< 0.001) and *B. bassiana *(*P *< 0.001, Tables 4 and 5) on mosquito survival declined over time regardless of the conidia concentration used. For *M. anisopliae*, survival of mosquitoes exposed to each concentration 1 d post application were lower than survival of mosquitoes exposed to similar concentrations 14 and 28 d post application (*P *< 0.001). No difference, however, was observed for mosquitoes exposed to each concentration between 14 and 28 d post application (*P >*0.05, Table 4). For *B. bassiana*, survival of mosquitoes exposed to each concentration 1 d post application was lower than survival of mosquitoes exposed to same concentrations 28 d post application (*P *< 0.001). No difference however, was observed for mosquitoes exposed between 1 and 14 d, as well as 14 and 28 d post application (*P *> 0.05, Table 5). Concentration did not tend to influence the decline in conidia persistence (*P *> 0.05, Figure 5). The MSTs of mosquitoes exposed to 2 × 10^9 ^conidia m^-2 ^of either isolate of fungus at all time intervals post application ranged between 11 - 13 d, while that of 2 × 10^10 ^and 4 × 10^10 ^ranged between 6 - 12 d. The survival of mosquitoes exposed to fungus was always lower than that of controls (*P *< 0.001, Tables 4 and 5). The MSTs of controls ranged between 15 - 16 d.

**Table 4:** Kaplan-Meier pair wise comparisons of the median survival times (MST) of *An. gambiae *s.s. females exposed to surfaces 1, 14 and 28 d after treatment with different concentrations (2 × 10^9^, 2 × 10^10 ^and 4 × 10^10 ^conidia m^-2^) of *M. anisopliae *ICIPE-30.

Comparison of:		Different concentrations within same time post-application			The same concentration between different times post-application	
			
	MST ± SE	2 × 10^9^	2 × 10^10^	4 × 10^10^	14 d	28
**1 d**						
Control	15 ± 0.95	χ^2 ^= 15.15	χ^2 ^= 43.34	χ^2 ^= 55.58	χ^2 ^= 0.08	χ^2 ^= 0.01
		*p *< 0.001*	*p *< 0.001*	*p *< 0.001*	*p *= 0.77	*p *= 0.32
2 × 10^9^	11 ± 0.51		χ^2 ^= 9.32	χ^2 ^= 15.44	χ^2 ^= 5.17	χ^2 ^= 4.10
			*p *< 0.001*	*p *< 0.001*	*p *< 0.001*	*p *< 0.001*
2 × 10^10^	6 ± 0.46			χ^2 ^= 0.001	χ^2 ^= 9.80	χ^2 ^= 8.95
				*p *= 0.98	*p *< 0.001*	*p *< 0.001*
4 × 10^10^	8 ± 0.39				χ^2 ^= 14.74	χ^2 ^= 18.55
					*p *< 0.001*	*p *< 0.001*

**14 d**						
Control	16 ± 1.15	χ^2 ^= 30.48	χ^2 ^= 58.07	χ = 49.02		χ^2 ^= 0.43
		*p *< 0.001*	*p *< 0.001*	*p *< 0.001*		*p *= 0.54
2 × 10^9^	12 ± 0.55		χ^2 ^= 6.59	χ^2 ^= 4.08		χ^2 ^= 0.06
			*p *< 0.001*	*p *< 0.001*		*p *= 0.80
2 × 10^10^	10 ± 0.64			χ^2 ^= 0.21		χ^2 ^= 0.49
				*p *= 0.22		*p *= 0.83
4 × 10^10^	10 ± 0.45					χ^2 ^= 0.003
						*p *= 0.96

**28 d**						
Control	16 ± 1.15	χ^2 ^= 35.94	χ^2 ^= 77.49	χ^2 ^= 62.29		
		*p *< 0.001*	*p *< 0.001*	*p *< 0.001*		
2 × 10^9^	13 ± 0.33		χ^2 ^= 11.22	χ^2 ^= 5.76		
			*p *< 0.001*	*p *< 0.001*		
2 × 10^10^	11 ± 0.49			χ^2 ^= 0.56		
				*p *= 0.46		
4 × 10^10^	11 ± 0.61					

*Significant at *p *< 0.05

**Table 5:** Kaplan-Meier pair wise comparisons of the median survival times (MST) of *An. gambiae *s.s. females exposed to surfaces 1, 14 and 28 d after treatment with different concentrations (2 × 10^9^, 2 × 10^10 ^and 4 × 10^10 ^conidia m^-2^) of *B. bassiana *I93-825.

Comparison of:		Different concentrations within same time post-application			The same concentration between different times post-application	
			
	MST ± SE	2 × 10^9^	2 × 10^10^	4 × 10^10^	14 d	28
**1 d**						
Control	15 ± 0.95	χ^2 ^= 5.73	χ^2 ^= 20.48	χ^2 ^= 16.50	χ^2 ^= 0.08	χ^2 ^= 0.01
		*p *< 0.001*	*p *< 0.001*	*p *< 0.001*	*p *= 0.77	*p *= 0.32
2 × 10^9^	11 ± 0.42		χ^2 ^= 7.03	χ^2 ^= 5.01	χ^2 ^= 0.61	χ^2 ^= 4.09
			*p *< 0.001*	*p *< 0.001*	*p *= 0.44	*p *< 0.001*
2 × 10^10^	9 ± 0.55			χ^2 ^= 0.24	χ^2 ^= 1.74	χ^2 ^= 6.36
				*p *= 0.63	*p *= 0.19	*p *< 0.001*
4 × 10^10^	9 ± 0.79				χ^2 ^= 0.09	χ^2 ^= 6.03
					*p *= 0.77	*p *< 0.001*

**14 d**						
Control	16 ± 1.15	χ^2 ^= 29.08	χ^2 ^= 52.46	χ^2 ^= 49.02		χ^2 ^= 0.43
		*p *< 0.001*	*p *< 0.001*	*p *< 0.001*		*p *= 0.54
2 × 10^9^	12 ± 0.44		χ^2 ^= 4.23	χ^2 ^= 7.04		χ^2 ^= 5.12
			*p *< 0.001*	*p *< 0.001*		*p *< 0.001*
2 × 10^10^	10 ± 0.67			χ^2 ^= 0.37		χ^2 ^= 1.79
				*p *= 0.54		*p *= 0.18
4 × 10^10^	10 ± 0.45					χ^2 ^= 0.40
						*p *= 0.53

**28 d**						
Control	16 ± 1.15	χ^2 ^= 47.61	χ^2 ^= 34.67	χ^2 ^= 55.76		
		*p *< 0.001*	*p *< 0.001*	*p *< 0.001*		
2 × 10^9^	13 ± 0.42		χ^2 ^= 7.56	χ^2 ^= 4.92		
			*p *< 0.001*	*p *< 0.001*		
2 × 10^10^	12 ± 0.54			χ^2 ^= 0.249		
				*p *= 0.11		
4 × 10^10^	12 ± 0.64					

*Significant at *p *< 0.05

## Discussion

These experiments were designed to provide preparatory information necessary for the use of oil-formulated entomopathogenic fungi in field-based mosquito control. Due to the expense and logistics involved in applying any insecticidal agent on a large-scale, it is essential to first define the concentration and time required to infect and kill mosquitoes. Information concerning persistence is also needed to determine re-application rates.

Low conidia concentrations and short exposure times can result in small infective doses that can be countered by immune responses. Insect responses to entomopathogens involve melanization, encapsulation and phagocytosis of invading fungal blastospores [19], but it is likely that these responses can be overcome at high concentrations. In these experiments it was found that for both fungal species concentration was positively correlated with mortality, and that the maximum and most rapid reductions in mosquito survival were achieved at concentrations of 2 × 10^10 ^conidia m^-2 ^and above. With well standardized production systems, formulations, application methods and delivery tools, concentration of 2 × 10^10 ^conidia m^-2 ^can be operationally amenable. The efficacy of *M. anisopliae *in terms of mortality against *An. gambiae *s.s. was slightly, although not dramatically, higher than that recorded by Scholte *et al *[20], and these differences may be related to the use of mineral oil to formulate the conidia, instead of sunflower oil. Additionally, the quality of the conidia batch, the method of application and the target species can also impact on the efficacy of fungal applications. For example, in this study the efficacy of these two fungal species against *An. gambiae *s.s. was comparable, yet when a similar range of conidial concentrations were tested against *Anopheles stephensi*, *B. bassiana *was found to be much more effective than *M. anisopliae *[12]. Overall, a similar positive relationship between conidial concentration and mortality, as observed here, is also evident in the published literature [12,20,21]; it is difficult to directly compare studies because of differences in fungal isolate, oil formulation, target species, bioassay protocols and units used to express conidial concentration.

When *M. anisopliae *and *B. bassiana *were applied as a co-formulation against *An. gambiae *s.s., neither an additive nor synergistic effect was evident. Similar results have been found when entomopathogens were evaluated in combination against arthropods other than mosquitoes [22-25]. The initial interaction between the two fungal species occurs at the point of mosquito exposure. Even if conidia of both species adhere to the mosquito cuticle, a competitive advantage would be gained if one of the fungi was faster to invade and colonize the mosquito haemocoel. Following colonization, the successful fungus could prevent other fungi from becoming established by competitive exploitation, limiting resource availability, actively synthesizing and releasing inhibitory metabolites or stimulating host immune responses [26]. Considering that exposure to the co-formulation had no additive effect, it is likely that the activity of one fungal species was partially or completely redundant. The complete absence of co-sporulation and the predominance of *M. anisopliae *suggest a competitive advantage of *M. anisopliae *over *B. bassiana*. Additive [27] and synergistic [28,29] effects of co-infection have been recorded for other entomopathogens at sub-optimal temperature regimes. Thus the possibility remains that fluctuating temperature and or relative humidity either in the laboratory or field may affect co-formulations of *M. anisopliae *and *B. bassiana*.

The length of time required for conidia to infect and kill mosquitoes is an important consideration for developing dissemination tools for field use. The exposure times tested in the current study were selected to represent realistic exposure periods. Mosquitoes may spend up to 15 min trying to enter a bed net [30] and after blood-feeding may rest on a surface for up to 24 h [31], though in areas of high bed net coverage it is likely that mosquitoes spend on average less than six hours inside houses [32-34]. In this study it was found that exposure times as short as 15 and 30 min were sufficient for conidia of both *M. anisopliae *and *B. bassiana *to infect mosquitoes and reduce survival. Similarly, no effect of increasing exposure time beyond 5 minutes up to 6 h on infection rates was found when *An. stephensi *were exposed to 2 × 10^9 ^conidia m^-2 ^of *B. bassiana *[12]. Increasing the exposure time beyond 6 h and/or concentration did increase mortality of *An. stephensi *[12] and other arthropods [35,36]. When the concentration tested against *An. gambiae *was increased, no effect of exposure time was observed, though only relatively short exposure times were tested (15 min - 6 h). However, longer exposure times (24 h, 48 h and continuous) of *An. gambiae *to *M. anisopliae *were tested by Scholte *et al *[20] and at high concentrations and no difference between the exposure times tested was observed. Formulations of either *M. anisopliae *or *B. bassiana *could, therefore, be used with dissemination tools/surfaces that target host seeking (short contact) as well as resting (long contact) mosquitoes.

Despite a general decline in the persistence *M. anisopliae *and *B. bassiana *against *An. gambiae *s.s., conidia were still pathogenic up to 28 d post application. During the current study, the conidia were stored under constant temperature (26°C-27°C) and RH (85-95%) thus it is unknown if fluctuating environmental conditions would affect the length of residual activity. A slight decline over time in the germination of conidia when applied in the field has been reported elsewhere [17], yet 63% of conidia remained viable after three weeks. Most importantly it was found that increasing the concentration of conidia did not increase the residual activity. Although the residual activity of fungi is short lived compared with traditional synthetic insecticides, it is comparable with other successful bio-insecticides such as *Bacillus thuringiensis *var. *israelensis *[37,38]. The MSTs values recorded during the persistence experiment for exposure immediately after drying (1 d) were lower than MSTs observed when similar concentrations were tested elsewhere in this study. The difference could have been due to variation in the quality of the conidia batch.

To be capable of transmitting malaria, a mosquito must survive for longer than the extrinsic incubation period of the pathogen, 9 - 14 d for *Plasmodium spp *[39]. This period is longer than the average mosquito life span and, therefore, malaria transmission can be attributed to a small fraction of the mosquito population. Daily survival is actually the most sensitive component of vectorial capacity [40,41] and thus control strategies that reduce vector age are highly desirable. The current study recorded large reductions in the daily survival of female *An. gambiae *s.s. when exposed to relatively high (2 × 10^10 ^and 4 × 10^10^conidia m^-2^) concentrations of both *M. anisopliae *and *B. bassiana*. These concentrations were lower than that used by Scholte *et al *[17]. When mosquitoes were exposed to high conidial concentrations in this and other studies [11,16,17,20], 100% mortality was often achieved within 10 d. If these results can be replicated in the field this could lead to a considerable reduction in malaria transmission [42].

## Conclusions

Of the few biological control tools targeting adult mosquitoes that are currently under development (including fungal, bacterial, viral and protozoan pathogens), entomopathogenic fungi are likely to be developed for programmatic use. Especially since fungus production and application all involve relatively simple infrastructures and processes, which could potentially be adopted in malaria endemic countries. An application of either *M. anisopliae *or *B. bassiana *at a concentration of 2 × 10^10 ^conidia m^-2 ^should be able to infect mosquitoes in a relatively short time (15 or 30 min) for up to one month after application. This concentration should provide a considerable safety margin for application error, exposure time and residual activity. However, there remains a need to test the fungi in large-scale field trials and to develop protocols to ensure simple and economical distribution and application in malaria endemic developing countries. Further developments to increase conidia persistence are still necessary in order to enhance the potential epidemiological impact of fungi on malaria transmission.

## Competing interests

The authors declare that they have no competing interests.

## Authors' contributions

LLM, TLR, MJK, DWL and MWM were directly involved in the data collection, analysis and drafting of the manuscript. BGJK and WT conceived the study, obtained funding for it and revised the manuscript before submission. All authors read and approved the final manuscript.

## Supplementary Material

Additional file 1 **Survival curves of *Anopheles gambiae *s.s. fungus treated and non-treated surfaces**. The survival of *Anopheles gambiae *s.s. females after exposure to 2 × 10^10 ^and 4 × 10^10 ^conidia m^-2 ^of *Metarhizium anisopliae *ICIPE-30 and *Beauveria bassiana *I93-825 for different times (15 and 30 min). Click here for file
